# Uncovering the Science-Policy Interface: Applying Bibliometric Approaches to the Wildfire Risk Management Domain

**DOI:** 10.1007/s00267-025-02161-x

**Published:** 2025-04-23

**Authors:** Juliane Schlierkamp, Claudia Berchtold, Isabelle Linde-Frech

**Affiliations:** Fraunhofer INT, Appelsgarten 2, 53879 Euskirchen, Germany

## Abstract

The impact of research is gaining increasing importance, as science is increasingly seen as a means to address humanity’s grand challenges. Consequently, interaction between science and policymakers is essential — a process formalized through Science-Policy Interfaces (SPIs). But who actually participates in these processes? This question is crucial, as scientific findings are not always consistent: they may be subject to interpretation, contradict each other, or be shaped by underlying normative frameworks. This paper explores the potential of bibliometric analysis to trace science-policy interactions, using the Wildfire Risk Management (WFRM) domain as a case study. Drawing on data from the Dimensions database, we examine publication and policy trends, disciplinary coverage, and the influence of Altmetrics on policy citations. Our key findings indicate that: There is a significant time lag (6–9 years) between scientific publication and policy adoption. The number of publications in a research field correlates with policy citations, but not all disciplines are equally represented in policy documents. Altmetrics, particularly social media attention, influence policy uptake, suggesting that visibility beyond academia plays a role in knowledge transfer. Data quality issues in linking scientific research to policy documents persist, limiting full traceability. Despite these limitations, the study highlights the potential of bibliometric approaches to support the development of more transparent and accountable SPIs. With improved data infrastructure, such methods could help policymakers better identify and integrate relevant scientific insights.

## Introduction: The Science- Policy Interface in WFRM

The impact of research is increasingly gaining importance in funding programmes such as Horizon 2020 and its successor Horizon Europe, which emphasize the need for communication strategies, stakeholder engagement, and policy outreach. This reflects a broader understanding of science as a means to address global challenges such as climate change and pandemics.

Policymakers play a crucial role in shaping transformation processes and defining the conditions under which research can generate societal impact. Accordingly, research projects today go beyond publishing in academic journals: they produce policy briefs, participate in advisory committees, and engage with stakeholders to inform decision-making — a process often referred to as the science-to-policy push. At the same time, a range of policy forums and mechanisms has emerged to pull scientific knowledge into policy, particularly in areas such as environmental governance^1^ - for example, through institutions like the Intergovernmental Panel on Climate Change (IPCC).

This process of interaction — commonly referred to as the Science-Policy Interface (SPI) – can be defined as “social processes which encompass relations between scientists and other actors in the policy process, and which allow for exchanges, co-evolution, and joint construction of knowledge with the aim of enriching decision-making” (van den Hove 2007). But who is actually participating in such processes? This question is on the one hand relevant since scientific findings are not necessarily coherent. Scientific findings may be interpreted differently, might contradict one another, or may be based on normative assumptions. On the other hand, complex challenges require the involvement of all relevant disciplines and if certain disciplines are not heard, policies might as well only address parts of the problem. For example, at the beginning of the COVID-19 Pandemic, virologists played a predominant role while it became clear that also sociologists, psychologists and economists must play a role to design holistic policies (Matthews 2020).

However, SPI processes often appear as black boxes that are difficult to reconstruct or understand from the outside. It is not necessarily clear who contributed to certain papers or who was involved in committees and fora according to which criteria. At the same time, Wildfire Risk Management (WFRM) is characterised by great complexity and cross-sectoral, multi-scalar challenges and is - due to the increasing impacts of fires – a range of new policies are being developed and a lot of public funding is made available to address these challenges.

To unpack these processes and review the scientific input in policy papers, this paper explores the potential of a bibliometric approach by applying bibliometric methods to trace and track science-policy interactions applying it to the field of wildfire risk management (WFRM) as an illustrative example of related to SPIs. It therefore initially provides some background to SPIs and governance challenges in the WFRM context. In a second step it details the methodology applied for bibliometric analysis and presents the results for the WFRM context.

### The Science-Policy Interface (SPI): Some Theoretical Grounding

#### Definition, changes over time and fields of application

The Science-Policy Interface (SPI) refers to the social interactions involving scientists and various participants in the policymaking process, facilitating the exchange, mutual development, and collaborative generation of knowledge to enhance decision-making (van den Hove 2007). SPIs have thereby not been static but evolved over time. More specifically, for the 20^th^ century, three main phases can be identified (Sokolovska et al. 2019):

i. the “linear phase” (1960s–1970s) when science informed policy-making in a unidirectional manner,
ii. the “interactive phase” (1970–2000s) when both sides found themselves in a continuous interaction, and
iii. the “embedded phase” (starting from the 2000s) when citizens’ voices come to be involved within this dialogue more explicitly.

Over time, the communicative relationship between science and policy-making has grown increasingly complex, with a growing number of actors and interests involved. In other words, SPIs cannot be characterised as simple knowledge transfer but have to be regarded as a complex relation between different institutional logics. Science thereby serves as a source of legitimacy in the policy process for advancing but also for delaying or avoiding action (Boehmer-Christiansen 1995).

The shaping and purpose of SPIs depends on the type of problem, phase in the policy cycle and the scale of the problem affecting for example the national or international level. Purposes of SPIs may thereby encompass the following fields (Engels 2005):

- *Warning* about a new or emerging risk or threat to human wellbeing that requires for an intervention
- *Problem definition*: understanding the causal relationship between sources and impacts also suggesting “what should be done, what can be done, by whom it can be done, and at what costs”
- *Impact assessment*: facilitating the choice among policy instruments
- *Monitoring and evaluation* of policy choices.

#### SPI Application

The use of scientific evidence in decision and policy making is not a new concern. The role of science in societal decision-making processes plays and increasing role since the early 1960s. Pioneering works such as E.M. Rogers’ “Diffusion of Innovations” (Rogers 1962) or Latour and Woolgar’s “Laboratory Life: The Social Construction of Scientific Facts” (Latour and Woolgar 1986) have shaped our understanding of how scientific knowledge is produced, disseminated, and translated into societal impact — often beyond its original disciplinary boundaries. (Boaz et al. 2019).

Today, the scientific insights are widely applied including for example health and healthcare, environmental and sustainability issues, international development, social care, education, or criminal justice to name just a few (Boaz et al. 2019). At the International level, organisations such as the Intergovernmental Panel on Climate Change (IPCC) or the intergovernmental science-policy platform for biodiversity and ecosystem services (IPBES) exemplify SPIs are strategically institutionalised to synthesise and translate scientific knowledge into actionable policy advice.

Today, many research funding systems that are responding to specific challenges have been established. For example, the European Union has structures its Horizon Europe research and innovation funding programme around five missions with the aim “to bring concrete solutions to some of our greatest challenges” (European Commission 2023):

1. Adaptation to Climate Change: support at least 150 European regions and communities to become climate resilient by 2030
2. Cancer: working with Europe’s Beating Cancer Plan to improve the lives of more than 3 million people by 2030 through prevention, cure and solutions to live longer and better
3. Restore our Ocean and Waters by 2030
4. 100 Climate-Neutral and Smart Cities by 2030
5. A Soil Deal for Europe: 100 living labs and lighthouses to lead the transition towards healthy soils by 2030

#### SPI Challenges

In all these domains, the interaction between science and policy making faces some challenges. van den Hove 2007 has, for example, discussed the *objectivity of knowledge*. Building on Popper’s distinction between subjective and objective knowledge (1979) she stresses that objective (scientific) knowledge “even though it strives to be objective, is always intertwined with subjective knowledge which is eminently social and political.” (van den Hove 2007) Secondly, it is not always clear if and w*hy a particular piece of expertise is used* while others remain ignored (Hisschemöller et al. 2018). This is specifically interesting since different scientific findings might be contradicting and advice may hence not necessarily be subject to (scientific) consensus. Thirdly, an increasing number of policy problems becomes cross-sectoral and increasingly complex which also requires for a more diverse scientific input. These challenges call for a traceability and trackability of scientific contributions to the policy debate to understand who are the scholars and individuals having a voice. On the contrary, it is important to understand which disciplines might not have been included in decision-making processes.

### Quality Criteria for SPIs: The Matter of Transparency

Against the described background, the evaluation of SPIs is part of a scientific debate. Jones et al. (1999) for example addressed the information transfer between science and policy, dealing with aspects of accessibility and relevance of research as well as receptivity of policy makers in the climate change context. Similarly, Heink et al. (2015) utilize and put into practice elements of knowledge trustworthiness, pertinence, and acceptability in Science-Policy Interfaces (SPIs) to evaluate their actual impact on policy, institutions, or processes. They have discussed challenges regarding the meaning of these terms, their ambiguous use and their applicability to assess effectiveness.

Respective works suggest important approaches to evaluating SPIs or more explicitly, the quality and effectiveness of SPIs in terms of scientific findings translating into decision making. As such they presuppose that interacting stakeholders are set but do not address the selection process as such (Boswell 2018). Acknowledging that SPIs are non-linear processes but take place in complex environments spanning multiple disciplines of the natural and social sciences, Gluckman et al. (2021) take up the idea of boundary functions guiding this process. They suggest the conceptual framework of *brokerage* at the science–policy interface, dealing with the complexity of mechanisms for requesting and receiving advice from the scientific community and connecting the communities in terms of aligning information needs with outputs and translating the different languages. SPI brokerage comes increasingly relevant since a wide range of science advisory mechanisms have evolved over the past decades. However, the COVID-19 pandemic has not only illustrated that they had to be complemented with ad hoc mechanisms but also that the evidence considered in these processes had been highly variable. While the involvement of social sciences is overall a rather recent phenomenon in advisory processes (Kropp and Wagner 2010; Matthews 2020), their consideration specifically during the beginning of the pandemic was modest, for example with respect to their input into early epidemiological models (Bertozzi et al. 2020; Metzler 2020).

The lacking consideration of certain scholars may hence lead to incomplete or (from a disciplinary point of view) even counterproductive or false recommendations. At the same time, these lackings pose a challenge to *transparency* as a core principle of democratic institutions. The right of the citizen to know what the government is doing and why (according to which insights and assumptions) as a key aspect of democracy can however only be accomplished if also SPIs and interaction between certain scholars and scientists are transparent.

Against this background, several options to increase the transparency on SPIs exist, which may include example registers on expert commissions and their members. One example at European level is Register of Commission Expert Groups which provides information on the Commission department which is running the group, as well as on its members, missions and tasks. Another option is the use of bibliometric data as provided by respective data bases which we want to explore further with this paper.

Within the scientific community, bibliometric data is an established tool to analyse and monitor scientific processes. In 1955 the Science Citation Index was published as a tool to facilitate the identification of relevant publications (Garfield 2007). In the early 2000s, when scientific web databases became widely accessible, several bibliometric indices, such as h-index, SciVal or InCites, were developed. Although there is criticism^2^, these data are a useful tool to handle the increasing publication numbers (Bar-Ilan 2008; Hicks et al. 2015). Since 2010 Altmetrics came up as alternative means to observe the evaluation of scientific work. These metrics consider the attention within and outside the scientific community by regarding not only citations in scientific publications but also citations in social media and news (Priem et al. 2010). Therefore, Altmetrics can be used to monitor the knowledge transfer on interfaces such as SPIs.

Besides to Altmetrics, platforms as Dimensions or Overtone also aim to enable the analysis of SPIs. Dimensions is a platform which was developed in 2018 and contains 127 million publications, 6 million grants, 11 million datasets, 200 million online mentions, 743 thousand policy documents, 695 thousand clinical trials and 145 million patents. (Dimensions o. D)

### Science Policy Interfaces in the Wildfire Risk Management (WFRM) domain

This paper addresses the reconstructability of SPIs in the context of Wildfire Risk Management by applying bibliometric analyses. It has been selected this field for four reasons that are explained in more detail in this section:

1. WFRM is characterised by great complexity and cross-sectoral, multi-scalar challenges.
2. It is gaining importance due to the increasing impacts of fires (partly) driven by climate change.
3. A dynamic in policies such as the development of new WFRM policies at national level, the need to share resources at European level to fight fires or the development of new policies and organisations to govern wildfire risk in countries heavily affected over the last years such as Portugal or Greece.
4. In line with the above-mentioned aspects, public funding has increased to address new challenges. To ensure that this funding is spent in effective and efficient ways, it needs to be ensured that the range of latest scientific insights should be coherently integrated into policy.

#### WFRM complexity

Wildfire risk and its management is characterised by complex interdependencies between human behaviour, socioeconomic development, climate, and the vegetation resources otherwise known as fuel (load) (Paton et al. 2015; European Science and Technology Advisory Group 2020). This holds for example true with respect to the interplay between climate and vegetation or antropogenic impacts on ignitions and fire propagation (see for example Duane et al. 2021). At the same time, the interplay between a range of socio-economic developments and interests and activities of different types of sakeholders also play an important role in shaping the overal risk. For example, unemployment, especially among young people, can lead to abandonment of traditionally cultivated landscapes and an increase in population size of cities, thereby on the one hand increasing fuel loads in the country and the extent of wildland-urban interfaces (WUI) on the other hand, as for example in the Western Mediterranean region of Europe (European Science & Technology Advisory Group 2020). Similarly, different institutions and organisations may have diverging interests, needs, policies and practices. For example, “one division within a natural resource agency may aim to restore ecological conditions and processes on historically fire-prone forestlands while another division will aim to suppress fire on those same lands” (Fischer et al. 2016). This can be problematic since fire exclusion over long periods allows for increased fuel density and contribute to the conditions for extreme fires. In Europe, traditional burning has ceased in many places (Rego et al. 2010), creating a paradox around the benefits of the use of fire and its impact on wildfire. In addition, responsibilities are not always aligned with the necessary resources. For example, in Portugal households sometimes lack the equipment to implement the preventive measures that they are entrusted with by law related to maintaining a clean perimeter around their houses. (European Civil Protection and Humanitarian Aid Operations 2019). Finally, WFRM also encompasses redistributive aspects. This became apparent after the 2018 wildfires in Sweden, where half of the burned area (6400 ha) mainly owned by small-scale private landowners was declared a nature reserve after the fire (Johansson and Lidskog 2020). These redistributions are a reaction on the increasing impacts of wildfires.

#### Increasing impacts

**Fig. 1:**
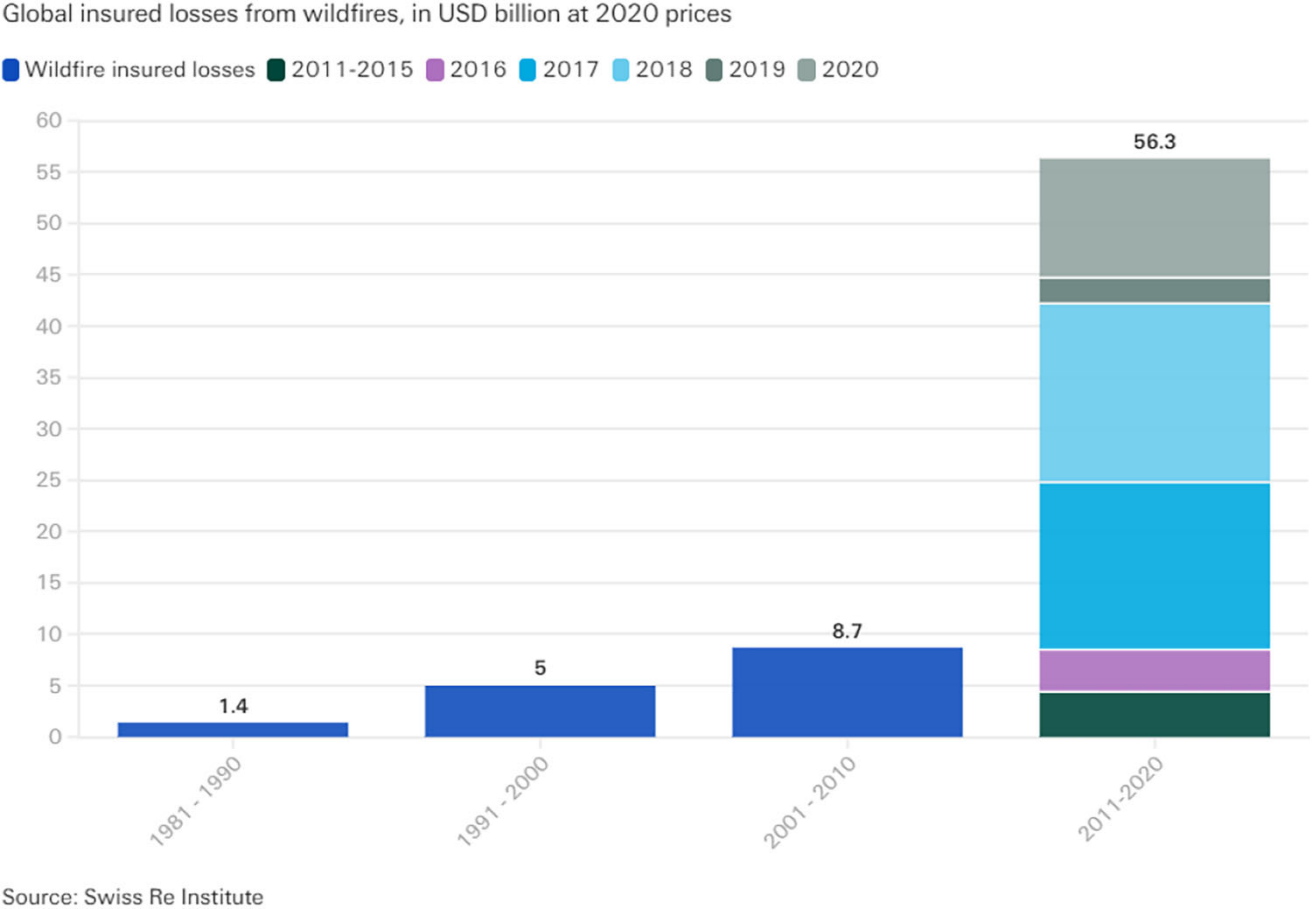
Global insured losses from wildfires, (Swiss Re Institute 2022)

In this complex environment, climate change as well as human activities are increasing the impacts of wildfires across the globe, which increases threats to human societies including goods and lives, as well as to investments, and planning or future economic activities (Duane et al. 2021; Bowman et al. 2017; McLauchlan et al. 2020) and “recent global events (2016 Canada, 2017 Chile, USA, and Portugal, 2018 Northern Europe, South Africa and the USA, 2019 Bolivia, 2020 Australia and USA) point to the emergence of novel extreme wildfire events not reported previously and associated, in many areas, to a higher frequency of events than expected” (Duane et al. 2021; Sharples et al. 2016; Boer et al. 2017; Paton et al. 2015; Tedim et al. 2018; Nolan et al. 2020). The number of losses is thereby drastically increasing as the figures of the Swiss Re Institute in the figure below show Figure 1 (Swiss Re Institute 2022). “In the last four years alone, wildfires were responsible for almost a quarter of all secondary-peril insurance losses worldwide. This is unprecedented: before 2016, the share of this peril in losses averaged just above 3% and rarely exceeded 5–10%” (Swiss Re Institute 2022).

#### Policy dynamics

In the changing landscape of managing wildfire risk, three main developments can be identified that underline the need for more transparent SPIs: new policies are designed at national level in very affected Mediterranean countries, in Central and Northern European countries, and at European level as detailed below.

##### Holistic policy design and governance restructuring at the national level in Mediterranean countries

After the devastating wildfires in Greece in 2018, an external expert committee reviewed the existing wildfire challenges and the civil protection system. The committee attributed shortcomings in effective prevention efforts within the existing Greek system, among others, a lack of common and coordinated approaches in fire prevention planning, occasional or very limited public information, awareness and mobilization, ineffective organization of volunteerism as well as the great discrepancy in the funds allocated to *prevention* in relation to the funds allocated to the suppression of fires. The report hence suggested

“a holistic approach, through an integrated and coherent framework for landscape fire management [to …] address jointly the prevention and suppression of fires, as well as the restoration of burned areas in an integrated manner to ensure and strengthen societal, environmental and economic resilience” (The Global Fire Monitoring Centre 2019)

Similar observations had been made in Portugal in 2017, where 2017 marked a year of new wildfire extremes in which more than 120 lives were lost (Turco et al. 2019), even though support was received through the EU Member States. Two long-term trends were identified as main drivers contributing to the massive impacts: the abandonment of rural landscapes that have become economically less relevant, as well as the dogma of fighting fire: land use change, together with efficient fire response to small fires led to an accumulation of vegetation, resulting in increased risks of large wildfires (Rego et al. 2007). In order to address the underlying factors of (rural) wildfire risk in an integrated manner, the creation of a dedicated authority (Agência para a Gestão Integrada de Fogos Rurais - AGIF) was enacted by resolution 157-A/2017 of the Council of Ministers (Conselho de Ministros 2017). AGIF was granted the authority to analyse, plan, evaluate and coordinate integrated rural fire (risk) management. Besides this risk specific umbrella competence, it is mandated to facilitate the integration of the communities by campaigns such as *Portugal Chama* to address and citizens and involve them into the risk management processes (Agência para a Gestão Integrada de Fogos Rurais 2020).

##### Arising threats and need for policy adaptation in Central and Northern European countries

Overall, European landscapes, especially the mosaic-like vegetation patterns in Mediterranean ecosystems, have been significantly influenced by a lengthy history of human land use, climate fluctuations, and related disruptions like fires. Yet, in contrast to this, Northern and Central European countries such as Sweden, Germany or the Netherlands historically have rarely had to deal with large wildfire events. The Swedish Civil Contingencies Agency (MSB) had even excluded wildfires from their 2013 catalogue of potential serious incidents and “the legal requirement for fire services to uphold a capacity to coordinate operations in response to large-scale incidents was not in active compliance throughout most of the country” (Bynander and Nohrstedt 2020). However, more countries suffered from large forest fires in 2018 than ever recorded before, including in Central and Northern Europe. Sweden even experienced its worst fire season requesting assistance via the Union Civil Protection Mechanism (UCPM) (European Environment Agency 2021).

Wildfire events are likely to become more frequent in Northern Europe since the meteorological fire danger^3^ is projected to increase further in most regions and “a northward expansion of moderate fire danger zones in western-central Europe” can be expected EEA (2021) building on (Rigo et al. 2017; Camia et al. 2017; Costa et al. 2020).

### WFRM Policy Changes at European Level

Due to this northward extension in wildfire danger and the increasing frequency and intensity of wildfire events, two developments are taking place at European level:

i. structures to share resources for response operations through the Union Civil Protection Mechanism (UCPM) have been strengthened (rescEU) and
ii. simultaneously, efforts for facilitating the development of integrated wildfire risk management (WFRM) policies are increasing.

The sharing of resources was strengthened through a revision of the legislative basis at EU level by Decision 2019/420, namely a revision of the Union Civil Protection Mechanism (UCPM) in 2019 with the objective of enhancing both the protection of citizens from disasters and the management of emerging risks. The rescEU reserve thereby establishes a new European reserve of resources which includes a fleet of firefighting planes and helicopters among others.

“*For the 2021 forest fire season, the European Commission co-financed the stand-by availability of a rescEU firefighting fleet to address potential shortcomings in responding to forest fires. Croatia, France, Greece, Italy, Spain and Sweden put together 11 firefighting planes and 6 helicopters at the disposal of other EU Member States in case of an emergency in exchange for financial contribution of the stand-by costs of these capacities.”* (European Civil Protection and Humanitarian Aid Operations n.a.)

In addition, the revision of the UCPM also requires for the development of a Knowledge Network. In addition, the European Parliament had initiated a preparatory action to study the feasibility of a Network of European Hubs for Civil Protection and Crisis Management. In its final report, structures for the creation of a Wildfire Knowledge Hub are suggested, also encompassing training formats on (joint) Command and Control structures as well as technical wildfire fighting practices including their integration potential into the existing UCPM Training programme structures (DG ECHO 2020). Respective undertakings are supported by the funding of civil protection projects under the Knowledge Network and connected via the online platform which went online in December 2021 (European Union n.a.).

#### Public research funding

In parallel, a broad range of research projects have been funded over the past decades. In total 56 EU WFRM projects receiving a contribution of 103.2 million € have been funded since the 6^th^ Framework Programme in different European funding schemes. (European Commission; Directorate-General for Research; Innovation et al. (2018), 23f). They addressed six thematic areas, namely fire science, prevention, suppression and post-fire recovery, integrated fire management as well as land, aerial and space detection Under the European Green Deal, aiming at no net emissions of greenhouse gases by 2050 and an economic growth decoupled from resource use (European Commission 2019) about additional 65 million € have been made available to “Preventing and fighting extreme wildfires with the integration and demonstration of innovative means” with the aim to accelerate “the shift towards implementing a more holistic fire management approach that integrates environmental, climate, health & safety/security, cultural and socio-economic aspects” as wildfires release greenhouse gases 4.

### Research Questions

As described above, it is not always clear how scientific findings are used in an inform policy development. This raises the question of whether there are biases that influence the transfer of knowledge. The following research question is to be answered:

Are there biases that influence the transfer of scientific findings to policy documents in the wildfire risk management research complex?

In order to divide and specify this question, the following subsequent research questions are posed:

1. Is there a time lag between the publication of scientific publications and the publication of the policy document? (RQ1)
2. Do more publications in a field of research lead to more citations in policy documents? (RQ2)
3. Are publications that are frequently mentioned online more often cited in policy documents? (RQ3)

- Are there any correlations or differences between the Altmetric Scores of the publications cited in policy documents and those of the publications as a whole?
- How are the Altmetric Scores of the publications cited in policy documents composed?

By dividing the main research question into these three subsequent questions, three levels are considered to identify potential bias. To answer the Research Question 1 the numbers of publications over time are considered. For the Research Question 2 the numbers of publications are clustered into *Fields of Research*. The research question 3 is considered on the level of the individual publications. By regarding these three levels, a systematic approach to the explorative main research question is developed.

## Methodology: Bibliometric Analysis

To quantitatively analyse knowledge transfer at SPIs, a bibliometric approach was applied. The study investigates whether systematic biases in the transfer of knowledge can be identified using a set of bibliometric indicators derived from scientific publications and policy documents.

As part of this approach, Altmetrics are introduced to assess the broader visibility and attention of publications. In addition, the development of the study’s hypotheses and the statistical tools employed for the analysis are described.

### Altmetrics

Altmetrics (*Alternative Metrics*) are indicators that reflect the attention a publication receives across both scientific and non-scientific contexts. The term was coined on Twitter in 2010 (Piwowar 2013) and emerged in response to the growing number of platforms for publishing and disseminating scientific results.

Traditional bibliometric indicators — such as citation counts or the Hirsch index — are often only meaningful several years after publication (Havemann 2016). Moreover, they are subject to systemic biases in scientific communication. These metrics tend to favour conventional research approaches and well-established publication channels, potentially slowing down innovation. They were also developed at a time when the volume of scientific publications was much lower than today.

Altmetrics, in contrast, are available shortly after publication and applicable to large numbers of documents. By incorporating attention from outside the academic community, they help reduce some of the biases inherent in traditional scholarly evaluation. For these reasons, Altmetrics are considered a valuable complement to conventional bibliometric indicators (Priem et al. 2010; Howard 2012; Galligan and Dyas-Correia 2013).

Several provider offer Altmetric services, including *Altmetric.com*, *PlumX Metrics* und *PLoS Article Level Metric (ALM)* (Franzen 2015). In this study, we focus on the Altmetric Score and the Altmetric Donut, both provided by Altmetric.com.

The Altmetric Score is a composite measure that captures attention across various platforms. It is calculated by summing the weighted counts of mentions a publication receives. The specific platforms and their respective weightings are listed in Table 1.

**Table 1:** Platforms and weighting factors for the Altmetric Score

Platform	Weighting factor
News	8
Blogs	5
Policy documents	3
Clinical guidelines	3
Patents	3
Wikipedia	3
Peer review	1
Weibo (until 2015)	1
Google+ (until 2019)	1
F1000	1
Syllabi (open syllabus)	1
LinkedIn (until 2014)	0.5
Bluesky	0.25
X (formally Twitter)	0.25
Facebook (public sites and profiles only)	0.25
Reddit	0.25
Pinterest (until 2013)	0.25
Q&A (stack exchange)	0.25
YouTube	0.25

### Hypothesis and Statistical Tools

To address the research questions using statistical methods, we derived a set of hypotheses based on the respective questions.

For Research Question 1 (RQ1), we test the hypothesis:

“There is no time lag between the publication of scientific articles and the publication of corresponding policy documents.”

This hypothesis is examined in two ways. First, a descriptive comparison of the publication timelines is conducted — comparing the number of scientific publications related to Wildfire Risk Management (WFRM) over time with the timeline of policy documents.

Second, the hypothesis is tested through multiple regression analysis. The regression model uses the number of scientific publications referring to WFRM over the past 14 years as independent variables to predict the annual number of policy documents. The resulting model thus includes 14 predictor variables (one for each year), 14 corresponding coefficients, and one constant:

$${n}_{{PD}}\left(t\right)={b}_{t-1}* {n}_{{pub}}\left(t-1\right)+{b}_{t-2}* {n}_{{pub}}\left(t-2\right)+{b}_{t-3}* {n}_{{pub}}\left(t-3\right)+{b}_{t-4}* {n}_{{pub}}\left(t-4\right)+{b}_{t-5}* {n}_{{pub}}\left(t-5\right)+{b}_{t-6}* {n}_{{pub}}\left(t-6\right)+{b}_{t-7}* {n}_{{pub}}\left(t-7\right)+{b}_{t-8}* {n}_{{pub}}\left(t-8\right)+{b}_{t-9}* {n}_{{pub}}\left(t-9\right)+{b}_{t-10}* {n}_{{pub}}\left(t-10\right)+{b}_{t-11}* {n}_{{pub}}\left(t-11\right)+{b}_{t-12}* {n}_{{pub}}\left(t-12\right)+{b}_{t-13}* {n}_{{pub}}\left(t-13\right)+{b}_{t-14}* {n}_{{pub}}\left(t-14\right)+\epsilon$$

Research Question 2 (RQ2) is addressed by testing the hypothesis:

“The number of publications per Field of Research (FoR) does not correlate with the number of citations in policy documents.”

To test this hypothesis, we analyse histograms and scatter plots showing the number of publications across FoRs and their citation frequency in policy documents. In addition, we apply a Spearman rank correlation test to assess the strength and direction of this relationship (Handl and Kuhlenkasper 2018).

To answer Research Question 3 (RQ3), we test the hypothesis:

“The number of online citations does not correlate with the number of citations in policy documents.”

This hypothesis is examined by comparing the Altmetric Scores of publications cited in policy documents with the scores of all publications related to WFRM. Key statistical measures — including mean, median, range, and standard deviation — are used to identify significant differences between both groups.

**Table:** 

**Level**	**Research question**	**(Null) Hypothesis**	**Tools**
Publications over time	Is there a time lag between the publication of scientific publications and the publication of policy documents which consider the findings from the scientific publication?	There is no time lag between the publication of scientific publications and the publication of the policy document.	Comparison of Timelines Multiple Regression
Publications per Field of Research	Do more publications in a field of research lead to more citations in policy documents?	The number of publications per field of research does not corelate with the number of citations in policy documents.	Histograms Scatter Plots Rank correlation test according to Spearman
Altmetrics	Are publications that are frequently mentioned online more often cited in policy documents? a) Are there correlations or differences between the Altmetric Scores of the publications cited in policy documents and those of the publications as a whole? b) How are the Altmetric Scores of the publications cited in policy documents composed?	The number of online citations does not correlate to the number of citations in policy documents.	Key figures (range, mean, standard deviation) Evaluation of the Altmetric Donuts

### Data Collection

Bibliometric data for this study were collected from the Dimensions database, a scientific research platform launched in 2018. Dimensions provides a wide range of interlinked publication types, including journal articles, books, conference papers, awarded grants, clinical trials, and policy documents. A key strength of the database lies in its ability to map citation linkages across publication types, such as citations of scientific literature in policy documents. Dimensions also classifies publications using a hierarchical taxonomy of 22 superordinate and 152 subordinate Fields of Research (FoR). Its data sources include Crossref, PubMed, and several Open Access platforms (e.g., DOAJ, OpenCitations, I4OC), as well as patents, policy documents, and Altmetrics. The platform aims to manage and present large-scale scholarly data in a transparent, structured, and user-friendly way. (Dimensions 2021; Hook et al. 2018; Singh et al. 2021)

To identify relevant literature on Wildfire Risk Management (WFRM), we developed a search query based on the research algorithm by Hirth and Nordhausen, using a building block approach. Terminology support was provided through Fraunhofer KATI^5^ to ensure comprehensive keyword coverage.

The data were retrieved from Dimensions on January 3, 2025, using the following query (applied to titles and abstracts):

(wildfire OR “forest fire” OR “wildland fire” OR “bush fire”) AND (prevent OR prepar OR response OR impact OR restauration OR restore OR adapt* OR manage* OR behave* OR fight*)

This query was designed to capture a broad spectrum of publications relevant to wildfire risk management and related intervention strategies. In total, the search yielded 174 policy documents and 30,069 scientific publications.

To complement this dataset, Altmetric Score compositions for the policy-cited publications were retrieved separately from Altmetric.com on January 10, 2025, to ensure consistency across data sources.

## Findings of the Bibliometric Analysis

In the following, we present the results of the bibliometric analysis of scientific publications and policy documents related to Wildfire Risk Management (WFRM). In addition, we report initial findings on data quality, particularly concerning the completeness and accuracy of bibliometric linkages.

### Publications Over Time

To address Research Question 1, we analysed the temporal distribution of publications related to Wildfire Risk Management (WFRM) over the period from 1970 to 2020.

A building block search approach was applied to identify relevant publications. To ensure comprehensive coverage of relevant terminology, the Fraunhofer KATI tool was used to refine and validate the search terms.

#### Descriptive statistics

**Fig. 2:**
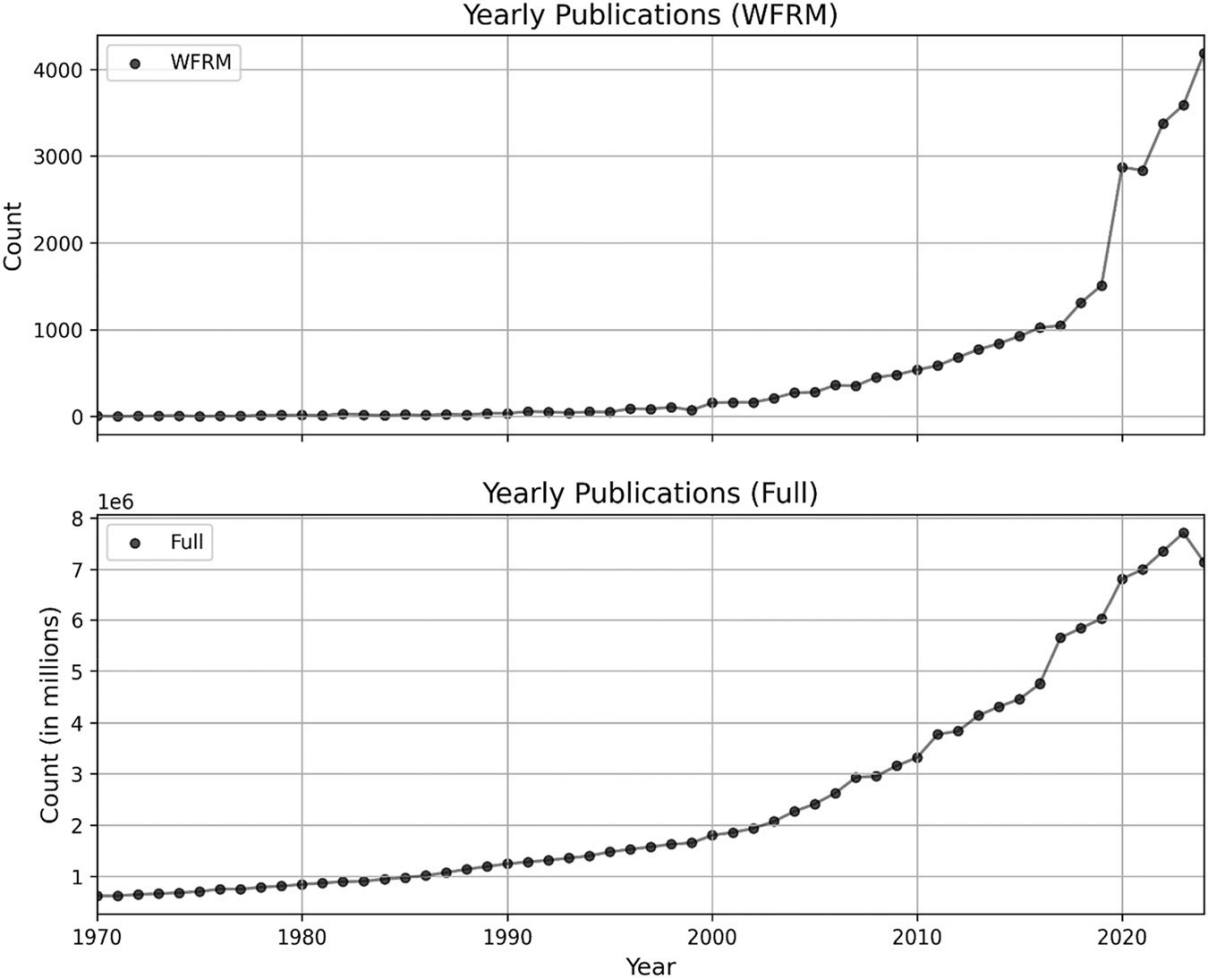
Annual number of publications

The number of publications related to Wildfire Risk Management (WFRM) has increased significantly over time. While fewer than 20 publications per year were recorded in the 1970s, the number rose to a peak of 4183 publications in 2024. This development is illustrated in Fig. 2, which also includes the total number of publications listed in the Dimensions database for the same observation period.

Both datasets reveal a noticeable trend shift around 2020, likely due to the general decline in scientific output during the COVID-19 pandemic.

From 2019 onward, the growth rate of WFRM-related publications diverges significantly from the growth trend of overall publications. This increase may reflect heightened awareness and urgency following major wildfire events in recent years. For instance, in 2015, North America experienced its most devastating wildfire season since record-keeping began (WWF 2020; Breitkopf 2020a). In Germany, 1071 wildfires were recorded that year — the highest number in over a decade and twice as many as in 2013 and 2014 (Breitkopf 2020a). The total area affected was 525.5 hectares, four times larger than in 2014, marking the most extensive fire damage since 2008 (Breitkopf 2020b).

A notable outlier is observed in 2006: while 278 publications related to WFRM were recorded in 2005, the number rose to a local maximum of 360 in 2006 before slightly declining to 352 in 2007.

To investigate the cause of this temporary increase, the content of WFRM-related publications for the years 2005, 2006, and 2007 was analysed in more detail (see Table 2). Based on this analysis, three distinct trends can be identified:

1. Increasing trend from 2005 to 2007:
  In USA, Australia, Italy, Germany and China the number of publications increased both from 2005 to 2006 and from 2006 to 2007. This numbers can declare the global growth from 2005 to 2006 but not the decline from 2006 to 2007.
2. Declining trend from 2005 to 2006, but increasing trend from 2006 to 2007:
  The numbers of publications decline from 2005 to 2006 in Greece an increase from 2006 to 2007 and therefore are contrary to the global trend. However, due to the small number of publications, this only has a very limited impact on the overall trend
3. Increasing numbers from 2005 to 2006, followed by declining numbers from 2006 to 2007:
  In Canada, Spain, United Kingdom, France and Portugal the national publications numbers correspond to the global trend.

**Table 2:** Annual numbers of publications by state

Nation	Number of publications			Trend
	2005	2006	2007	
USA	102	156	158	↑
Canada	21	39	34	↑↓
Spain	13	27	25	↑↓
Australia	16	21	23	↑
Italy	11	16	20	↑
United Kingdom	11	21	12	↑↓
Germany	6	14	15	↑
France	9	12	8	↑↓
China	7	9	12	↑
Greece	6	5	7	↓↑
Portugal	2	10	6	↑↓

One possible explanation for the significant increase in publications between 2005 and 2006 is the major heatwave in 2003 in Southern Europe, which triggered extensive wildfires across northern Italy, Corsica, Sardinia, Portugal, and south-western Spain (Lyamani et al. 2006). In Germany, a total of 2524 wildfires were recorded in 2003 — five times more than in both the preceding and following year. It was the highest number of wildfires in Germany since 1992, and remained unmatched until 2019 (Breitkopf 2020a). The affected area was twice as large as the combined areas impacted in the years 1997–2002. Wildfires in 2003 were not limited to Europe. In Victoria, Australia, an area of 1.3 million hectares was affected — the largest wildfire event in the country since 1939(Lane et al. 2006).

Overall, the number of publications related to wildfires follows the general trend of exponential growth in scientific output (Parthey and Biedermann 2002). However, notable deviations from this trend can be linked to major wildfire events. Based on the data, a time lag of approximately three years can be observed between significant wildfire occurrences and subsequent increases in publication numbers — suggesting a delayed but tangible response within the scientific community.

To assess whether this scientific activity translates into policy uptake, we next analysed the temporal development of policy documents. According to Dimensions, a total of 2,367,087 policy documents were published between 1970 and 2024. Of these, 134 documents include references to Wildfire Risk Management (WFRM). A marked increase in WFRM-related policy documents is evident between 2010 and 2015, reaching a local maximum of 15 documents in 2015 (see Fig. 2, diagram below). From 2015 onward, the number of such documents fluctuated between 2 and 12 per year, showing no clear upward trend. The first WFRM-related policy document was published in 1978 by the World Meteorological Organization in Switzerland, followed by another in 1992 from the United Nations Economic Commission for Europe. Since 2000, a gradual increase in WFRM policy publications can be observed, although annual figures remain variable.

**Fig. 3:**
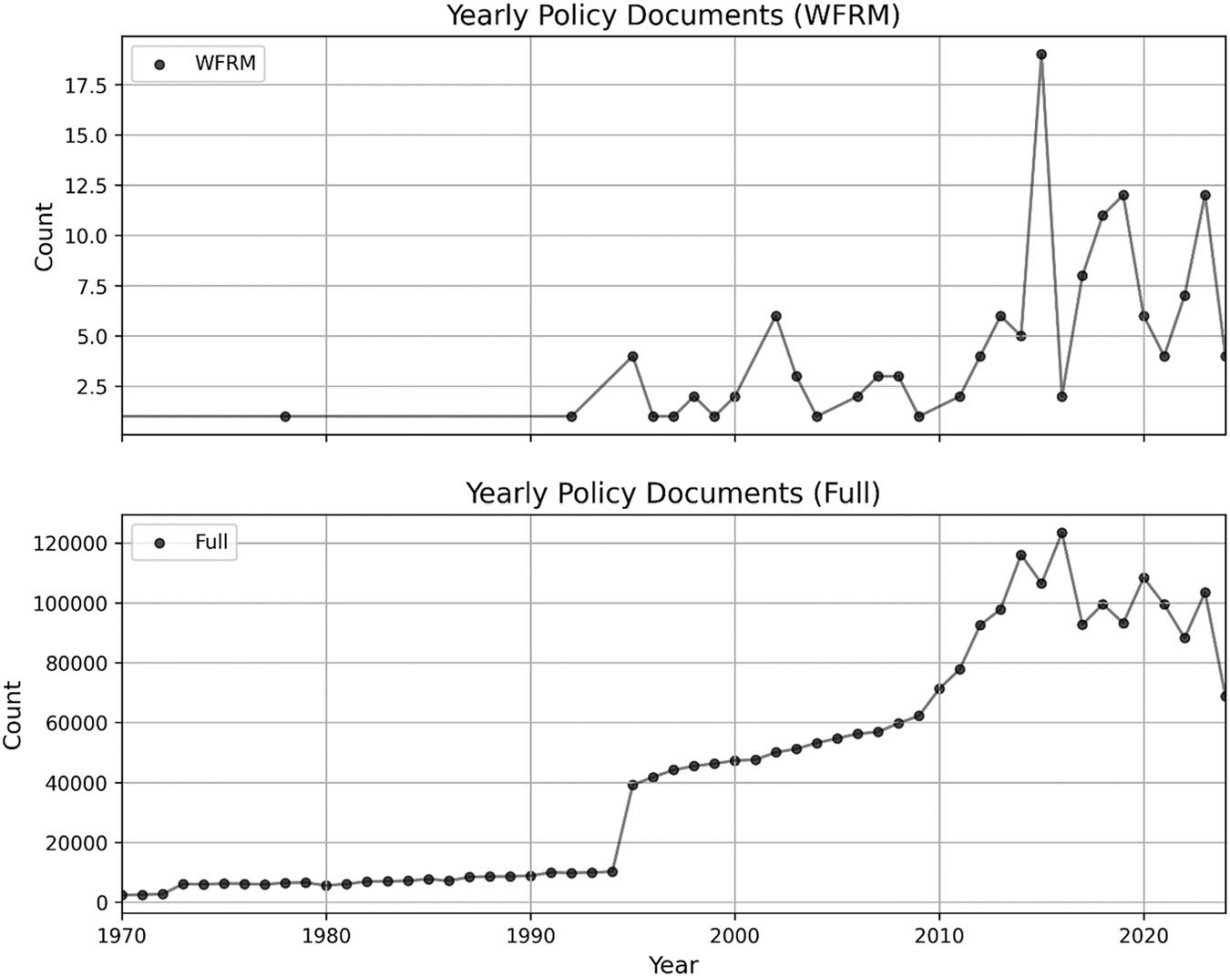
Annual numbers of policy documents

Interestingly, the overall number of all policy documents also peaked in 2015, followed by a period of volatility and general decline. Thus, while a broader upward trend from 1995 to 2015 is evident, both general and WFRM-specific policy publication rates have shown strong fluctuations since then Fig. 3.

To further examine the relationship between WFRM-related scientific output and corresponding policy documents, a multiple regression analysis was conducted.

The model includes data from 1990 to 2024 and uses the number of annual scientific publications related to WFRM as independent variables. Specifically, the model incorporates the publication dynamics of the previous 10 years to estimate their influence on the number of policy documents in a given year. Accordingly, the regression includes 10 lagged predictors (coefficients) and one constant.

The regression yielded a statistically significant result with a *p*-value of 0.0787 and an R² value of 0.618, indicating a moderate to strong explanatory power of the model.

**Fig. 4:**
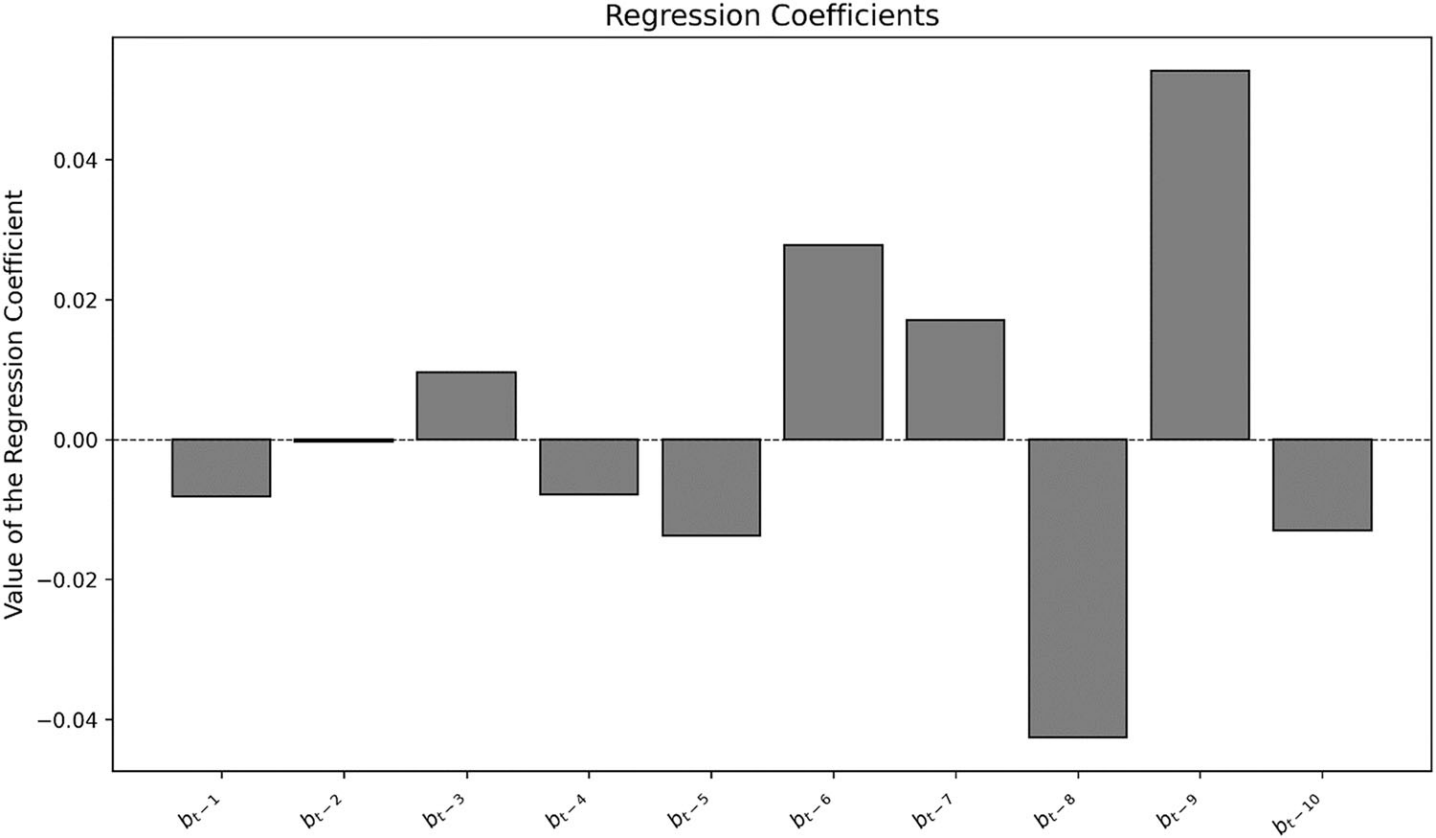
Bar chart of the regression coefficients of the regression model

In the Bar Chart in Fig. 4 illustrates the coefficients of the multiple regression model. Approximately half of the coefficients are positive, while the remaining half are negative.

The coefficients b_t-9_, b_t-6_ and b_t-7_ exhibit the strongest positive effects, whereas b_t-8_, b_t-5_ and b_t-10_ have the lowest (negative) values (Table 3).

### Fields of Research

Publications related to Wildfire Risk Management (WFRM) are distributed across a wide range of scientific fields. In total, 21 out of 22 superordinate Fields of Research (FoR) include publications with reference to WFRM. Among those publications that are cited in policy documents, 18 superordinate and 62 subordinate FoRs are represented Table 3.

**Table 3:** Regression coefficients of the regression model

Ranking	Coefficient	Value
1.	b_t-9_	0.0527
2.	b_t-6_	0.0278
3.	b_t-7_	0.0170
4.	b_t-3_	0.0096
5.	b_t-2_	−0.0003
6.	b_t-4_	−0.0078
7.	b_t-1_	−0.0081
8.	b_t-10_	−0.0130
9.	b_t-5_	−0.0137
10	b_t-8_	−0.0426

**Fig. 5:**
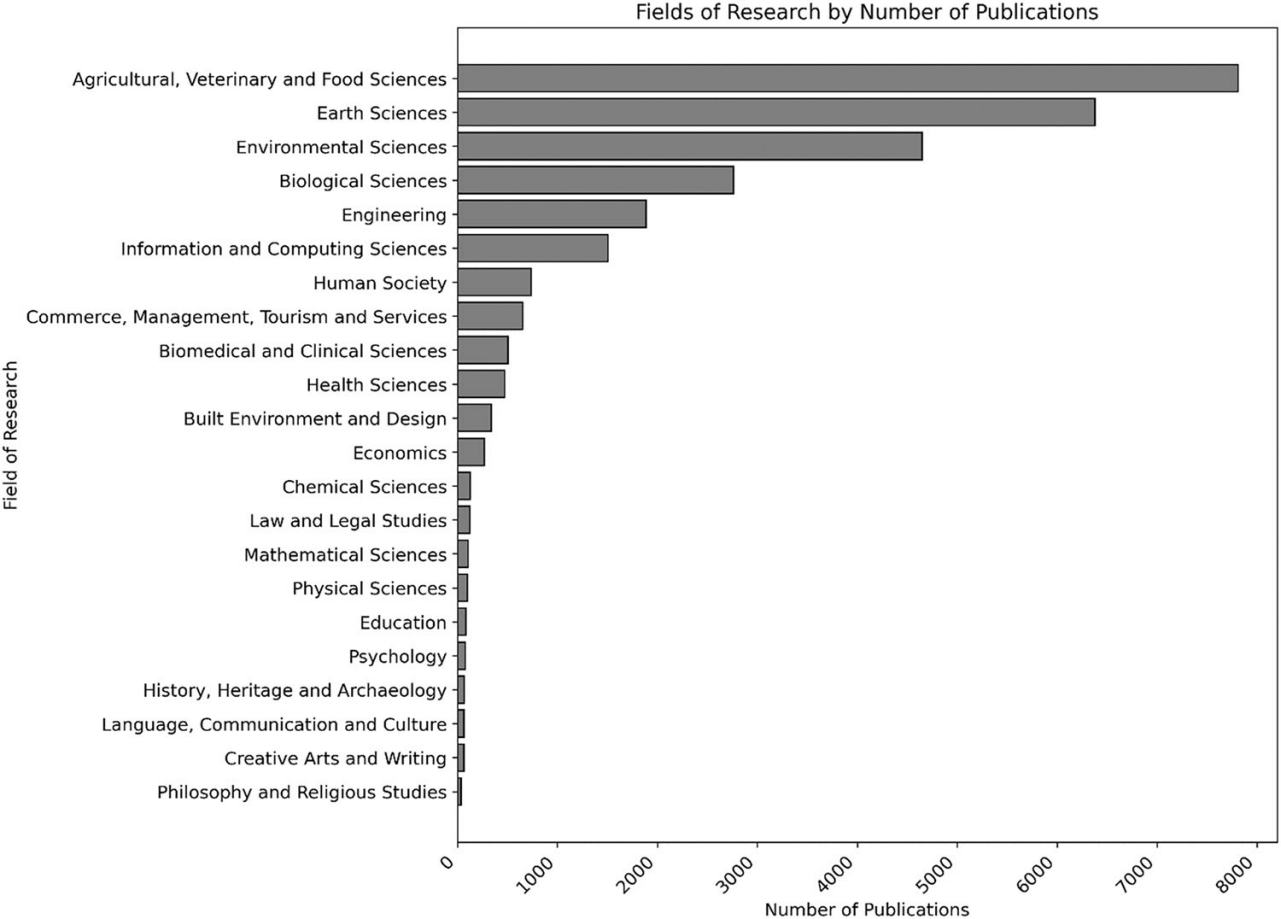
Numbers of publications per FoR

The Fields of Research with the highest number of WFRM-related publications overall are shown in Fig. 5:

- *Environmental Sciences:* 12.427 publications
- *Agricultural, Veterinary Sciences and Food Sciences:* 7.810 publications
- *Earth Sciences:* 7.162 publications
- *Biological Sciences:* 3.905 publications
- *Engineering:* 3.031 publications

Publications cited in policy documents related to WFRM can be assigned to 19 different superordinate Fields of Research (FoR). The Fields of Research with the highest number of policy-cited publications are:

- *Environmental Sciences*: 223 publications
- *Earth Science:* 147 publications
- *Health Sciences*: 13 publications
- *Agricultural, Veterinary and Food Sciences:* 96 publications
- *Biomedical and Clinical Sciences:* 90 publications

**Fig. 6:**
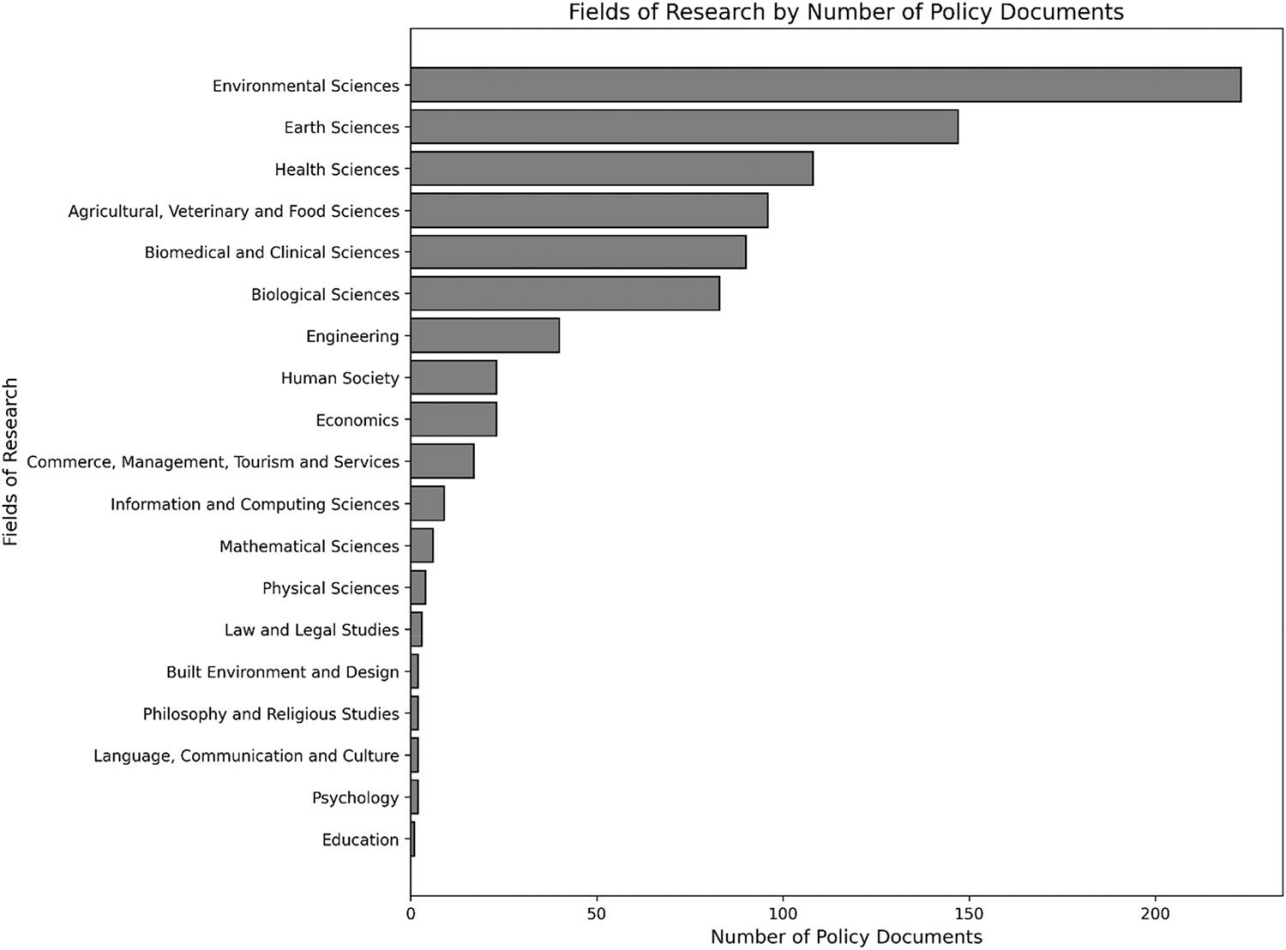
Numbers of publications per FoR cited in Policy Documents

When comparing the Fields of Research (FoR) of all WFRM-related publications with those cited in policy documents, several parallels become apparent. In both cases, *Environmental Sciences* represent the dominant FoR in terms of publication volume. *Earth Sciences* as well as *Agricultural, Veterinary and Food Sciences* also rank highly in both datasets. However, a notable difference emerges: publications cited in policy documents are more frequently associated with the health and medical sector, particularly *Heath Sciences, Biomedical and Clinical Sciences*. In contrast, the overall WFRM publication landscape shows a higher proportion of works from *Biological Sciences* and *Engineering*, indicating that these technically oriented disciplines are less represented in policy citations despite their strong publication output Fig. 6.

**Fig. 7:**
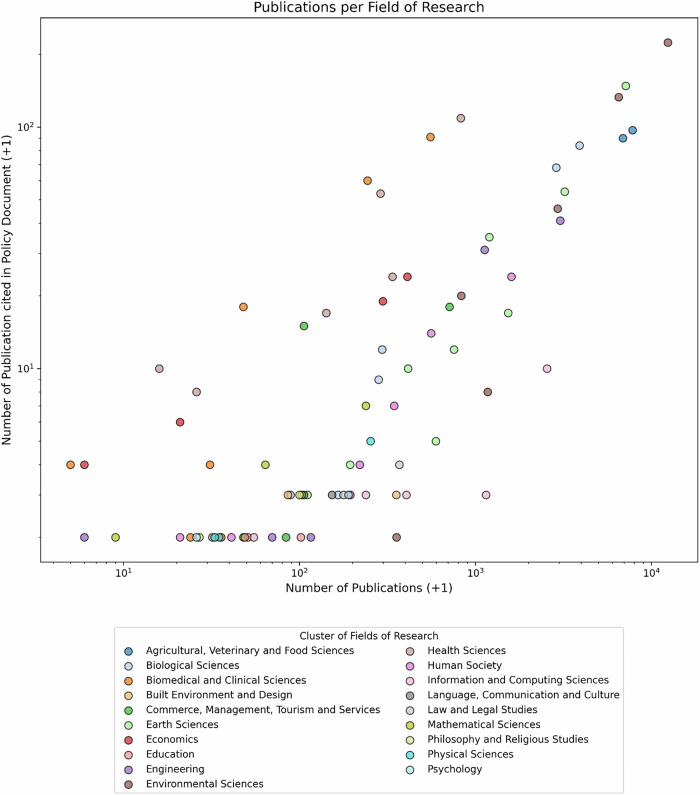
Number of publications per FoR, total and cited in policy documents

The scatter plot in Fig. 7 visualises the correlation between the total number of WFRM-related publications and the number of those cited in policy documents, with each dot representing a single Field of Research (FoR). Due to the wide range of publication counts per FoR, a logarithmic scale was applied. To avoid zero values for cited publications, all values in that dimension were increased by +1. The plot reveals a positive correlation: FoRs with higher numbers of total publications generally also show higher levels of policy citation. However, this relationship is not uniform across all fields. Notably, *Health Sciences* and *Biomedical and Clinical Sciences* exhibit a disproportionately high number of policy citations relative to their total number of WFRM-related publications. In contrast, other fields — such as *Information and Computing Sciences* — show relatively low policy citation rates, despite a substantial publication output.

In the next step, the relationship between the total number of publications and the number of policy-cited publications per Field of Research (FoR) was tested using the Spearman rank correlation.

The analysis reveals a statistically significant correlation (p = 1.098 × 10^−15^), with a correlation coefficient of ϱ = 0.74, indicating a strong effect according to Cohen’s classification (Cohen 1988).

### Altmetrics of the Publications

For the subset of publications cited in policy documents related to WFRM, both the Altmetric Scores and Altmetric Donuts^6^ were analysed.

The Altmetric Scores of all WFRM-related publications range from 1 to 13,189, resulting in a total range of 13,188. In contrast, the range of scores for policy-cited publications is 4792. The arithmetic mean of Altmetric Scores is 60 for cited publications, compared to 36 for the overall WFRM dataset. The median score is also higher among cited publications (7) than for the full set (4). Interestingly, the standard deviation is higher for the policy-cited group (263) than for the overall publication set (200), suggesting a greater variability in attention among the cited publications Table 4.

**Table 4:** Keyfigures of the Altmetric Scores

Value	Total of publication with reference to WFRM	Cited publication with reference to WFRM
Arithmetic mean	36	60
Median	4	7
Standard deviation	200	263
Range	13188 (xmin = 1; xmax = 13,189)	4792 (xmin = 3; xmax = 4795)

Since the Altmetric Score primarily reflects the overall volume of attention a publication receives, we also analysed the Altmetric Donuts to gain insight into the sources and platforms where this attention originates. For this purpose, we considered only those policy-cited publications with an Altmetric Score of 8 or higher. This subset consists of 254 publications, of which:

- 133 are cited on X (former: Twitter)
- 110 are cited in Blogs
- 47 are cited on Facebook
- 15 are cited on YouTube
- 9 are cited on Reddit
- 7 are cited on Google+
- 3 are cited on Bluesky
- 2 are cited in the Stack Overflow Q&A

Besides of the citations on social media, the publications are cited on:

- 254 in News
- 78 in Wikipedia
- 29 in Patents

### Interpretation of the Results

This chapter discusses the findings from sections 3.1.1, 3.1.2 and 3.1.3 in relation to the research questions and hypotheses.

In the analysis of the publications over time (3.1.1) suggests that wildfire events may be positively associated with an increase in WFRM-related publications. Peaks in publication numbers were observed in 2006 and again between 2018 and 2020, while major wildfire events occurred in 2003 and 2015. This indicates a potential lag of approximately three years between the occurrence of wildfire events and a corresponding response in scientific output.

With respect to policy uptake, a local maximum in the number of policy documents occurred around 2015, suggesting a possible time lag of 7–9 years between scientific activity and its reflection in policy. This is further supported by the multiple regression analysis, where the coefficients b_t-9_, b_t-7_ and b_t-6_ showed the strongest effects, again pointing to a lag of 6–9 years. Accordingly, the hypothesis

“There is no time lag between the publication of scientific publications and the publication of the policy document”

is rejected. A systematic time lag of approximately 6–9 years can be identified.

The analysis of Fields of Research (3.1.2 revealed indicators of a correlation between the number of publications in a given FoR and their frequency of citation in policy documents. While some FoRs showed higher or lower citation rates than expected, a general correlation is visible (Fig. 7). This finding is supported by the Spearman rank correlation, which yielded a statistically significant result (*p* < 0.001) and a correlation coefficient of ϱ = 0.74, indicating a strong relationship. Thus, the hypothesis

“The number of publications per field of research does not correlate with the number of citations in policy documents”

is also rejected. However, the fact that ϱ is not close to 1 indicates that publication volume is not the only influencing factor; other elements (e.g. visibility, relevance, or framing) likely play a role.

The Altmetric Scores of policy-cited publications are notably higher than those of the general WFRM publication set. Both the mean and median Altmetric Scores are elevated, and the narrower range of cited publications indicates that this difference is not solely driven by outliers. Additionally, Altmetric Donut analysis shows that publications cited in policy documents tend to receive more attention in social media, particularly on platforms like Twitter/X. These findings suggest that greater online visibility — especially via social media — increases the likelihood of policy citation. Therefore, the hypothesis

“The number of online citations does not correlate with the number of citations in policy documents”

is likewise rejected.

## Discussion

This paper has examined the role of Science-Policy Interfaces (SPIs) in complex policy-making processes, using Wildfire Risk Management (WFRM) as an illustrative case.It highlighted the importance of transparency in SPIs, particularly with regard to their role in supporting democratic decision-making. However, achieving such transparency remains a major challenge. The exponential growth in scientific publications — combined with a still substantial volume of policy documents, even though their numbers have declined since 2015 — makes it increasingly difficult to assess science-policy interactions using purely qualitative methods.

The findings of this bibliometric study are subject to several limitations. First, the results are specific to the domain of Wildfire Risk Management (WFRM) and cannot be directly generalized to other policy sectors. To draw broader conclusions about Science-Policy Interfaces (SPIs), the methodology should be applied to additional use cases across diverse domains. Second, the number of policy documents in the dataset is relatively low, and most were published after the year 2000. Given that our findings suggest a knowledge transfer lag of 6–9 years, this results in a limited observation window for analysing long-term science-policy dynamics.

**Fig. 8:**
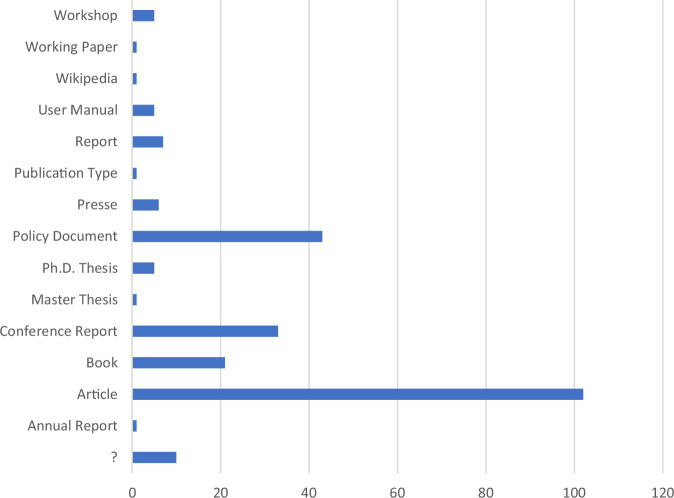
Sources of references in the exemplary analysis which are not listed in dimensions

Another important limitation concerns the quality and completeness of the underlying data. To assess this, a manual analysis of citations was conducted using the policy document “Advances in Remote Sensing and GIS Applications in Forest Fire Management” (EU Law and Publications) as a test case. According to Dimensions, this document includes 203 citations. However, a manual review identified 445 actual citations. One reason for the discrepancy of 242 missing citations is that Dimensions does not index certain publication types, such as workshop proceedings, working papers, or some conference reports. Notably, 112 of the 242 missing citations were peer-reviewed journal articles, which should be indexed by Dimensions (Fig. 8). Of these, only 18 articles were not found at all in the database; the remaining 94 were present in Dimensions, but not correctly linked to the policy document. This may be due to inconsistencies or incomplete references in the policy document itself, as different citation styles and formatting errors were observed. Another contributing factor could be the limitations of the AI-based citation recognition system used by Dimensions, which may be less effective in parsing policy documents, as these are not the platform’s primary focus.

A further limitation is that key policy documents are missing entirely from the database. For example, the influential EU report “Forest Fires: Sparking Fire-Smart Policies in the EU” is not included in Dimensions, despite its high relevance for WFRM.

The increasing availability of bibliometric data and the development of new analytical methods open up promising opportunities for reconstructing science-policy relationships. As demonstrated in this study, bibliometric approaches can also help identify the researchers and institutions influencing policy design — based on citations found in policy documents. This, in turn, offers a way to detect potential gaps or biases in the integration of scientific expertise.

In our case study, we analysed bibliometric data based on citations in WFRM-related policy papers indexed in the Dimensions database. The analysis relied on the reference lists provided by Dimensions for each policy document. However, as discussed above, these lists may be incomplete or inconsistent, as illustrated in the case study presented in Fig. 8.

A key challenge of this approach, therefore, lies in the quality and accuracy of automatically extracted citation data, particularly regarding policy documents. Improving the reliability of citation linking in bibliometric platforms will be essential for future applications of this method in studying Science-Policy Interfaces.

In addition to this shortfall, an analysis of the policy papers listed for the field of WFRM revealed two addition challenges:

i. Not all policy papers are listed. For example, an important one for the European level, namely “Forest Fires – Sparking firesmart policies in the EU” (European Commission; Directorate-General for Research; Innovation et al. 2018) was not included in the database. It was not possible to say whether this relates to EC publications only or why/how this shortfall can be explained. In essence though, we have to assume that the list of policy papers in Dimensions in incomplete. Further analysis and investigations would be needed to understand this gap.
ii. Only policy papers listed in English can be found via Dimensions. While this pre-selection seems reasonable, it also means that any related analysis can only relate the policy papers published in English. The suggested approach can hence not be applied to documents published in local language and hence the national level. This is a major shortfall, particularly when looking at the EU with several member states and native languages.

The use of bibliometric data to reconstruct Science-Policy Interfaces (SPIs) and to identify underrepresented disciplines in specific policy discourses offers significant potential. This is particularly true given its scalability and applicability across a wide range of disciplines. Bibliometric analysis could be used strategically to monitor the influence of particular research fields on policy over time — a promising but still underexplored methodology. However, the current limitations of bibliometric databases, such as incompleteness and citation extraction errors, must be acknowledged. As shown in this study, the automated listing of citations in platforms like Dimensions is often flawed. As a result, the insights generated are likewise partial and potentially biased. Other platforms, such as Overton, may offer more reliable data, but this would require further comparative analysis.

While the above limitations call for a cautious interpretation of the results, we argue that the core findings are still informative — especially since the identified biases likely affect all disciplines to a similar extent. Therefore, general patterns, such as the positive correlation between scientific publication volume and policy citation, may still be considered valid.

Moreover, our findings suggest that visibility in social media can enhance a publication’s likelihood of being cited in policy documents. Although the reference lists in Dimensions may be incomplete, the available data show that over 40% of policy-cited publications are mentioned on X (formerly Twitter), and 67% appear on at least one social media platform (X, Facebook, Google+, Bluesky). This indicates that social media attention plays a measurable role in knowledge transfer to policy.

Finally, we identified clear indicators of a 6–9-year time lag in the transfer of scientific knowledge into policy documents within the WFRM domain. Given the relatively short observation period, this finding should be confirmed through further studies and additional use cases.

## Conclusion

The use of bibliometric data and analyses offers considerable potential for understanding and reconstructing Science-Policy Interfaces (SPIs) at a broader scale. One direct benefit is the ability to compare the representation of different disciplines in policy processes. For complex challenges such as Wildfire Risk Management (WFRM), this may help broaden policy perspectives by highlighting valuable yet underrepresented disciplinary inputs.

Indirectly, bibliometric transparency can contribute to strengthening democratic principles by enhancing accountability in science-policy relations. Especially in times of disinformation, “fake news”, and growing science scepticism, such an approach could improve credibility and trust in both scientific evidence and political decision-making.

The insights generated in this study must, however, be viewed in light of the incompleteness of policy document indexing and citation data in current databases. Assuming these limitations affect all research fields equally, we may still draw meaningful conclusions — for example, that not all Fields of Research (FoRs) are equally considered by policymakers. Disciplines such as Earth Sciences, Engineering, or Ecology contribute to WFRM through scientific publications but appear underrepresented in policy documents. This information can help policymakers identify disciplinary blind spots — or conversely, highlight where scientists may not be communicating their findings effectively.

This paper also raises several follow-up research questions. First, the systemic errors in the citation data — likely stemming from automated extraction methods using AI and machine learning — are not yet fully understood. Greater transparency on the part of database providers would be needed to analyse these issues in depth. As access to policy citation data is typically commercially licensed, we expect that data quality will become a focus of future development.

Second, applying this approach to national or local-level policy documents could offer valuable insights into SPIs at different governance levels. However, such documents are currently not indexed by major databases, which makes them inaccessible for bibliometric analysis. Future developments should aim to close this gap.

## Data Availability

The data utilized in this paper was sourced from Dimensions.ai. All relevant data is accessible at: https://zenodo.org/records/14618273?token=eyJhbGciOiJIUzUxMiJ9.eyJpZCI6ImEwNjNiM2U1LTMwZDktNDM0MS1iMWY1LThjZWI3MjgzYWRiMiIsImRhdGEiOnt9LCJyYW5kb20iOiIwZWQ4YmQ0OWU0ZDAxMDRkZTFiMTkwYmQ1ODliZTdmOCJ9.H-cQUOFkeZqJAyK0jRtz7szTHBOVcoo32NdZIpCtRIczIo0xysSH4jzWT2y-5yIsp9YjeaauQfTCwsDby8epLQ. The Python scripts used for data analysis are available at the following repository: GitHub Repository: https://github.com/HackPaws/Uncovering-the-science-policy-interface.

## Footnotes

1 A bibliometric analysis shows that after „Studies in Human Society” and “Policy and Administiration” “Environmental Sciences” and “Environmental Science and Management” are the FoR with most results for Science-Policy Interface publications.

2 *Impact-factor obsession* describes a tendency in research of a soaring interest in only one crude measure Hicks et al. 2015.

3 The season with weather conditions furthering the potential of wildfires.

4 Preventing and fighting extreme wildfires with the integration and demonstration of innovative means | Programme | H2020 | CORDIS | European Commission (europa.eu)

5 Fraunhofer KATI is a bibliometric tool, of which one function is to identify key words for querys.

6 The altmetric donut shows the distribution of the number of citations on different platforms
